# Clinical Lecture on Fissure of the Anus

**Published:** 1841-09

**Authors:** 


*Clinical Lecture on Fissure of the Anus.*—By M. Velpeau. The patient who now occupies bed No. — is affected with fissure of the anus, a disease which still requires for its elucidation careful research. Notwithstanding the labors of Boyer, Beclard, Dupuytren, and a few other surgeons, I believe that we have much to learn of the causes, symptoms, and treatment of this painful affection; I shall, therefore, embrace the present opportunity of directing your attention to this subject.

Fissure of the anus consists in the existence of a small narrow ulceration seated in the radiating folds at the margin of the anus, and usually attended with very excruciating pain, whenever the patient goes to stool. Before Boyer's time this disease was almost entirely unknown, and if we examine what has been said concerning it by other writers who preceded him, we shall be convinced that our knowledge of its causes, symptoms, &c., dates within the last twenty-five or thirty years.

Let us first turn our attention to the *causes* of fissure of the anus. This disease may be excited by constipation, piles, the evacuation of hard fæcal matter, in large masses, by mechanical injury, from the end of a lavement apparatus, for example, &c.; but in many cases it is developed without our being able to discover or trace any probable cause. It arises, in most instances, in a very gradual manner, and soon assumes the characters of other ulcerations about the part; hence we find great difficulty in assigning to it its true and efficient cause; nor do I think we could produce the disease by artificial means. It exists in individuals of both sexes, but attacks

*Ingleby on Uterine Hemorrhagy, p. 249.
†Thesis, Strasbourg, Feb. 1830.
‡Revue Medicale, t. iii., p. 22, 1830.
§Revue Medicale, *id.* ||*Ibid.*

females more frequently than males; it commonly appears between the ages of twenty-five and sixty, but has been observed at the ages of eighteen or twenty. I have seen it in a young man eighteen years of age, and in a girl of twenty-one; children, however, seem to be exempt from this affection. One of the most remarkable points connected with the history of fissure of the anus, is the great pain and suffering by which it is accompanied; effects which we are unable to reconcile with the slight degree of organic injury constituting the complaint. M. Blandin and M. Harvey de Chegoin think that fissures seated above or below the sphincter ani are of an insignificant nature, and heal of their own accord, or under the most simple treatment; while other fissures are attended by the symptoms described by Boyer. This seems to me to be a speculative distinction, for I have seen several cases of fissure of the anus in which violent pain was a prominent symptom, although the affection did not implicate the sphincter muscle of the anus.

One circumstance, gentlemen, connected with the history of this disease, merits particular attention. The sphincter ani muscle is in a state of permanent constriction. But is this a cause or an effect of the disease? Boyer asserted that this constriction formed a principal exciting cause, since division of the sphincter muscle immediately calms all suffering, without any application having been made to the fissure itself. On the other hand, MM. Roche, Sanson, and Blandin contend that the constriction of the sphincter is a result of the complaint, because the ulceration may frequently exist without our being able to discover it. We cannot, it is true, deny that fissure may sometimes exist without constriction, altho' it be accompanied by the signs of the latter affection, such as violent pain, burning heat, &c., on going to stool. I have witnessed several examples of this; but who can affirm there is no fissure, merely because the surgeon who examines may be unable to find one? The great authority of Boyer is the only cause of our attaching any weight to his assertions, for they have never been confirmed by the result of *post mortem* examinations. Perhaps, however, there may be some means of reconciling opinions on this interesting point in the history of fissure of the anus. Thus, we can understand how a small fissure, being irritated by the passage of stercoraceous matter, may excite spasmodic constriction in the muscular bands underneath it; and again, we can believe that strong spasmodic contraction of the anus, by inducing costiveness, may induce excoriation of the skin about the anus, and thus become a cause of fissure. Under this point of view, constriction of the sphincter ani and fissure are two distinct affections which are independent, but have a strong tendency to merge one into the other.

The *symptoms* of the disease are the following; the patient first experiences some pain on going to stool, and for some time after an evacuation from the bowels. The pain gradually increases in intensity and period of duration, and when it has arrived at its maximum, the patient suffers the most excruciating torture. The sensation excited by the passage of fæces is compared by the patient to the pain occasioned by a red hot iron, or to the tearing asunder the margin of the anus, and brings on a feeling of faintness or threatening of convulsions. In the intervals between each stool, the patient merely feels some lancinating pain or scalding, with a sense of weight about the part, and colic. As the time for evacuating the bowels approaches, the pain is manifestly increased, is most violent during the moment of expelling the fæces, and then gradually declines for a few hours. It occupies a circumscribed space about the margin of the anus, and is often attended by pulsation of the vessels like that which accompanies phlegmonous inflammation. The bowels now become obstinately constipated, and evacuations take place every eight or ten days, unless purgatives or clysters be employed. The patient feels such a dread of going to stool, that he defers the moment as long as possible, although he knows that such conduct will aggravate his sufferings. One patient at the Hotel Dieu, was heard to exclaim that he wonld rather die than go to the water-closet, so great was the pain during evacuation of the bowels. Some persons who labour under this disease have recourse to curious methods of avoiding the inconveniences occasioned by the passage of fæcal matter; thus, Boyer mentions the case of a lady who kept a canula constantly fixed in the rectum, to prevent the suffering which she experienced at each evacuation. But fluid stools, or even the passage of air, will occasionally excite very severe pain. Some patients are able to walk about, sit down, or attend to their ordinary business during the intervals between the attacks, but others are compelled to keep their beds in spite of the increased heat and pain thus occasioned. The shooting pains extend towards the bladder or uterus, and in some cases to the hypogastric region. The digestion is impaired; the patient eats little, to avoid the necessity of going to stool; he becomes thin and of a yellow hue; the face is expressive of suffering, and on looking at it, one would say that the patient labored under some severe organic disease; occasionally the slightest movement will excite the accesses of pain; coughing, spitting, blowing the nose, speaking or singing will aggravate it; any excess in eating or drinking will have a similar effect. The presence of the catamenia, also, increases the sufferings of women. The pain is at once excited by introducing any body into the rectum, as the pipe of a syringe, &c., and when we attempt to pass up the finger, we not only occasion severe agony, but feel that it is powerfully grasped by the constrictor muscle of the anus.

On separating the folds of the anus and drawing the rectum gently down, we perceive, at the bottom of some one fold, a small ulcerated fissure about one or two lines broad, by four, eight, ten, or twelve in length. The edges of the fissure are generally free from hardness; they are of a bright red color, and bear the strongest resemblance to the cracks which so often exist in the hands, feet, or corners of the mouth. A very small quantity of pus may be discharged from the fissure, and in some cases a little blood. It often happens that we find it extremely difficult to discover the fissure, hidden as it is in the folds of the anus, which is usually, in such cases, more or less of a funnel shape; we must carefully unfold the integuments, and desire the patient to make a few expulsive efforts. The fissure is then exposed; it may occupy any point of the margin of the anus, may scarcely reach the edge of the mucous membrane, or may be confined to the parts above the sphincter. In many cases the fissure seems to commence from or terminate in a pile; and usually it extends in a vertical direction upwards. We can often assure ourselves of the existence of this lesion by the mere touch; the instant the finger comes in contact with the fissure, the most violent pain is experienced by the patient, and we feel a hard, wrinkled cord, which indicates the precise situation of the crack. Such, gentlemen, are the ordinary symptoms of fissure of the anus; severe burning pain at the moment of going to stool; narrow, elongated and superficial ulceration at the edge of the anus; and violent contraction of the sphincter muscle.

*Progress of the disease.*—Fissure of the anus does not present all the symptoms now enumerated, from its commencement; it begins with very slight pain, or itching; tickling feel or creeping sensation, with heat after each stool. These symptoms may continue for six months, or even a year, before they become sufficiently severe to excite much attention. In other cases, the disease will acquire its greatest degree of intensity in a few weeks, or may be very severe from the commencement. You are not, either, to imagine that the series of symptoms already described will present itself in every case; many patients feel no pain whatever: others (and the patient on whom I am about to operate is of this class) suffer severe and almost constant pain. Our patient has a long fissure, with grayish, irregular, and granulated base, resting on indurated cellular tissue. She suffers much from burning pain whenever she goes to stool; the anus contracts violently, and it is with the utmost difficulty that we can introduce a canula, or even the tip of the little finger. You should therefore remember that Boyer's description of fissure of the anus is merely a general one, and that several varieties may present themselves to us, in which the symptoms described by Boyer do not exist, and to which the treatment recommended by him would be inapplicable.

*Treatment.*—Some cases of painful fissure may get well of their own accord. I knew a medical student who labored under this disease for seven or eight years, and then recovered without operation or any treatment. A few days ago I saw a patient in town who equally got well without any treatment, after three or four years. However, patients are in general very anxious to adopt some means of relieving their sufferings, and removing the unpleasant disease under which they labor. This may be effected with or without operation. Several ointments have been employed for this purpose. Boyer obtained a cure in one case by throwing into the rectum two or three spoonfuls per day of an injection composed of hog's lard, walnut juice, sorrel juice, and almond oil, of each four ounces; but here the fissure was attended with slight contraction of the sphincter.

M. Descudé informs us that we can cure the disease with large doses of the oleum hyosciami, and topical applications of mercurial ointment. Some surgeons speak highly of *douches* of cold water, of decoctions of chœrophyllum or poppy heads, &c. In a few cases I have succeeded with white precipitate ointment. Dupuytren employed with excellent effect, the following ointment, introduced into the anus by means of a tent: extract of belladonna, two drachms; lard, two ounces; honey, two ounces. More recently some practitioners have spoken in very favorable terms of *monesia*, and one or two cases of cure by this remedy have been cited; but I fear that it will soon meet the fate of most of our new remedies, which flourish for a time and are heard of no more. When the above mentioned means have been tried (and we must often try them to satisfy our patients) without success, we must have recourse to one of the following, viz., cauterization, dilatation, division of the sphincter muscle, or excision of the fissure. The disease is sometimes cured in the most satisfactory manner by running a stick of nitrate of silver over the whole surface of the fissure. Beclard pretends that he never failed in this way; but other surgeons have not met with the same success; I have tried it myself in many cases without obtaining any benefit, and think that the cases which presented themselves to Beclard, must have been slight, and unaccompanied with contraction of the sphincter muscle; besides, Beclard employed dilatation at the same time. Cauterization can only cure fissure of the anus by modifying the ulcerated surface, and transforming it into a simple wound, which heals like common solutions of continuity. In this way we explain the success obtained by Guy de Chauliac, and Dionis, who cauterized or scarified the ulcers, and by Guerin, &c., who applied the actual cautery, or irritated the surface of the sore with the nail, &c.

*Dilatation*, by the introduction of tents of lint graduallv enlarged, into the rectum, has often been attended with the best effects in the hands of Beclard, Dubois, Marjolin, and others. I have employed this mode of treatment with success in some cases, but it is often tedious and painful. In order to shorten the period of pain and diminish its violence, we should employ the largest tents that we can introduce into the rectum. The pain is at first very severe, but as soon as we get to the fourth or fifth tent it is much mitigated; the tents may be covered with any of the ointments which I have already mentioned to you. However, I should remark that the composition of the ointment does not seem to have any effect whatever. I have tried them all, and afterwards common cerate, and found the latter to answer as well. Dilatation then is the chief element of cure in such cases, and I believe that considerable success would attend its use if we could induce our patients to resist the pain which it, in the first instance, always occasions.

*Incision* of the sphincter ani was proposed by Boyer, and recommended by him as the best, indeed the only mode of treating fissure of the anus; his practice has been followed by most of our surgeons up to the present day. Boyer regards this method as infallible, yet several practitioners mention cases in which it has failed. He was naturally led to advocate this mode of practice, because he believed that contraction of the sphincter ani was the chief cause of fissure.

The preparatory steps of this operation are exactly the same as those for fistula in ano. The lower intestines are to be emptied by means of a lavement, or some mild purgative, in order to insure quietude for some time after the operation. The instruments employed are a straight, probe-pointed bistoury; a common bistoury; a large tent; a T bandage, and all the minor accessories. The patient is placed on the edge of a bed, with the head low, the under limb extended, the upper one flexed, and the buttocks kept widely apart by assistants. The surgeon now introduces the index finger of his left hand into the rectum, guides along it the flat side of his probe-pointed bistoury, and divides the sphincter. Should the fissure occupy the median line in front, he must not cut upwards, for fear of injuring the urethra or vagina. Boyer thought it sufficient to divide the sphincter at any point, without caring where the fissure may be; but I am of opinion that you will do well to pass the blade of the knife through the fissure at the same time that you divide the muscle. When this has been accomplished, you continue the incision upwards and downwards for an inch or two, so as to cut thro' the whole thickness of the sphincter. A single incision is usually sufficient; but if there be several fissures, or if excessive contraction of the sphincter be present, then we must make a second incision on the opposite side of the anus. When the edges of the fissure are rounded off, hard and thickened, we seize them with a forceps, and remove the hardened portions either with the knife or scissors.

The dressing is very simple. A tent of lint covered with cerate is placed between the lips of the wound, but its upper extremity must reach about an inch beyond the superior angle of the incision. The space between the buttocks is then filled with lint, and the whole supported with a T bandage. The tent must be supplied every day until cicatrization takes place. I have said nothing of the occurrence of hemorrhage, for it is almost impossible that any such accident should happen; but were it to arrive, you must have recourse to the usual means of arresting it, with which you are all familiar.

Such, gentlemen, are the methods of treatment generally employed for the cure of fissure of the anus; cauterization, dilatation, incision. The latter treatment is successful in a vast majority of cases; but as some modern practitioners have insisted on the point, that constriction of the anus is the effect, not the cause of fissure, their opinions have produced some new ideas relative to the treatment of this affection. Upon the principles of these gentlemen, I have practised excision of the fissure instead of dividing the sphincter muscle. This operation had been highly spoken of by Mothe and Guérin; I had mentioned it myself in 1832, and some of my operations were published in 1836. The following is the method which I adopted. The patient is placed in the same position as for incision of the sphincter; the point of the border of the anus occupied by the fissure is then seized with a hook, and a couple of strokes of the bistoury on the right and left complete the excision of the fissured part. Sometimes I emyloyed the scissors to remove it, but always avoided cutting the muscular tissue underneath. The operation is soon over, and unattended with pain; I have performed it eight or ten times at least, and have almost always succeeded in curing the patient. In one or two failures I was unable to find out whether the want of success depended on my not having cut out all the diseased structure, or on some other cause. I believe that when the complaint is of long standing, we should divide the sphincter, and at the same time remove the ulceration; that is to say, combine incision and excision together. I shall do this in the case we are about to operate on. The disease here has existed for several years; the ulcer is large, with a grayish, lardaceous base; and it is very probable that simple incision of the muscle would fail to effect a cure. You may ask me, perhaps, why I employ excision, and do not remain content with division of the muscle, a mode of treatment which has been sanctioned by experience. My reasons are the following. Division of the sphincter is an easy and quick operation, and attended almost certainly with success; but it compels us to cut through the deeper-seated tissues beyond the muscle. The wound which results always suppurates for some time, and may occasion dangerous accidents. The inflammation and formation of matter may extend to the pelvis and compromise the patient's life. I have seen two cases in which the patients died after a division of the sphincter for fissure of the anus. The operation of excision is entirely free from this danger, because the cellular tissue beyond the sphincter is not touched. The resulting inflammation is very slight, and the wound requires to be dressed for three or four days only. Finally, it is an operation much more simple than division, and one which we should always prefer in recent cases; but when the disease is of long standing, and the contraction of the sphincter violent, we should combine the two operations, so as to ensure success in the most obstinate cases.—*Provincial Med. and Surg. Journal*, April 3, 1841.

